# 

**Published:** 1922-05

**Authors:** 


### HOSPITAL and HEALTH

REVIEW

*Established 1886* No. 1850. *Vol. LXXI.*

*New Series.* Vol. 1. No. 8 *May* 1922 Price Sixpence

CONTENTS.

PAGE

NOTES AND COMMENTS :—
The British Medical Association and the Voluntary Hospitals—Sir Patrick Manson—The Hospitals Combined Appeal—The European Epidemic Menace—Remote Effects of Dental Disease—Lunacy Reform—Mental Hygiene—Auto-Suggestion—Poisoning by Marking Ink—The International Committee on Disablement—The Smoke Evil—A Warning to Testators, &c. . . . . . 209

THE DECLINE AND FALL (?) OF TUBERCULOSIS.—II. . 217
By Edgar L. Collis, M.A., M.D., M.R.C.P.

SAFEGUARDING THE MILK SUPPLY . . . . . 221

By Wilfred Buckley, C.B.E.

MILK PURITY : THE DISTRIBUTOR'S VIEW . . . 222
An Interview with Sir Reginald Butler

PUBLIC HEALTH AND EMIGRATION . . . . . 223

By Colonel G. T. K. Maurice, C.M.G.

ARE DOCTORS BECOMING UNPOPULAR ? . . . . 224

By R. W. Harris

APPROVED SOCIETIES AND HOSPITAL TREATMENT . 225

KING EDWARD'S HOSPITAL FUND : ANNUAL MEETING AND REPORT . . . . . . 227

REVIEWS . . . . . . . . 229

HOSPITAL FINANCE . . . . . . . 233

HOSPITAL NEWS . . . . . . . 235

CORRESPONDENCE. RECENT APPOINTMENTS. . . . 237

PARLIAMENTARY QUESTIONS AND ANSWERS . . . 239

